# The effect of youths as change agents on cardiovascular disease risk factors among adult neighbours: a cluster randomised controlled trial in Sri Lanka

**DOI:** 10.1186/s12889-019-7142-1

**Published:** 2019-07-08

**Authors:** Nadeeka Chandraratne, Miwa Yamaguchi, Susantha Indrawansa, Nalika Gunawardena, Keisuke Kuwahara, Zobida Islam, Yohei Kawasaki, Tetsuya Mizoue, Diyanath Samarasinghe

**Affiliations:** 1 Ministry of Health, Nutrition and Indigenous Medicine, Suwasiripaya, No. 385, Rev. Baddegama Wimalawansa Thero Mawatha, Colombo 10, Sri Lanka; 2 Department of Epidemiology and Prevention, National Centre for Global Health and Medicine, 1-21-1 Toyama, Shinjyuku, Tokyo, 162-8655 Japan; 3 The Foundation for Health Promotion, No.21/1 Kahawita Road, Attidiya, Dehiwala, Western Province 10350 Sri Lanka; 4 World Health Organisation Country Office for Sri Lanka, 5 Anderson Road, Colombo, Sri Lanka; 5 Teikyo University Graduate School of Public Health, Tokyo, Japan; 6 Biostatistics Section, Clinical Research Center, Chiba University Hospital, 1-8-1 Inohana, Chuo-ku, Chiba-shi, Chiba, 260-8677 Japan; 7 Department of Psychological Medicine, University of Colombo, Colombo, Sri Lanka

**Keywords:** Blood pressure, Body weight, Community adults, Randomised control trial, Youths, Sri Lanka

## Abstract

**Background:**

Mobilising non-professional health workers has been successful in improving community health, but the effectiveness of an education program targeting youths in a community-based approach remains unclear. The objective of this study was to investigate the effect of an intervention with youth on cardiovascular disease risk factors of community adults.

**Methods:**

A 12-month cluster randomised trial was conducted in a semi-urban area of Colombo in Sri Lanka. Facilitators trained youth club members aged 15–29 years to assess cardiovascular disease risk factors and take actions in the community to address relevant issues. The control group received no intervention. Body weight and blood pressure as primary outcomes and lifestyle of adults as secondary outcomes were measured pre- and post-intervention. Multilevel linear and logistic regressions were used to assess the effects of the intervention on changes in continuous and binary outcomes, respectively, from baseline to endpoint.

**Results:**

Of 512 participants at baseline, 483 completed the final assessment after the intervention. Regarding primary outcomes, the intervention group showed a significantly greater decrease in body weight after intervention than the control group. The mean (95% confidence interval) difference of body weight change for intervention versus control group was − 2.83 kg (− 3.31, − 2.35). There was no statistically significant difference in blood pressure between the two groups. Turning to the secondary outcomes, in diet, the intervention group had a higher probability of consuming at least one serving/day of fruits (*p* = 0.02) and a lower probability of consuming snacks twice/day or more (*p* < 0.001) than the control group.

**Conclusions:**

An intervention employing youths as change agents was effective in lowering body weight among community adults in Sri Lanka.

**Trial registration:**

Trial registration number: SLCTR/2017/002, Name of registry: Sri Lanka Clinical Trials Registry, Date of registration: 19th January 2017, Date of enrolment of the first participant to the trial: 1st February 2017.

**Electronic supplementary material:**

The online version of this article (10.1186/s12889-019-7142-1) contains supplementary material, which is available to authorized users.

## Background

Non-communicable diseases (NCDs) are the leading global cause of death and are responsible for 70% of deaths worldwide [1]. In Sri Lanka, NCDs account for 75% of total deaths, and 40% of these NCDs are cardiovascular diseases (CVD) [2]. Regarding the risk factors of NCDs in Sri Lanka, the prevalence of overweight and obesity is 18.9 and 3.5% in men and 32.9 and 10.0% in women [3], respectively, and the prevalence of high blood pressure is 30.5% in men and 26.2% in women [2]. Unhealthy behaviours such as lack of physical activity, unhealthy diet, tobacco use, and the harmful use of alcohol are common, which in turn may lead to obesity, raise blood pressure, and ultimately develop NCDs [1].

Middle-income countries in the east Asia and Pacific region will face workforce shortages of health professionals (i.e. physicians and all other health workers) because their demand will exceed supply due to economic growth, rapid population growth, and aging [4]. Several intervention studies have suggested an important role of non-professional healthcare workers, including community health workers, to combat NCDs in low- and middle-income countries [5–7]. A few intervention studies provided training programs for school-aged children to act as change agents for the improvement of their parents’ lifestyle [8–10]. In Sri Lanka, the promotion of healthy habits initiated by school children led to the reduced body weight and increased physical activity of their mothers [9]. A study in Brazil reported a reduction of CVD risk factors among parents of children who underwent an educational program for health promotion [8]. One study in China indicated that an education programme for primary school children was effective in lowering the salt intake of their families [10]. These approaches are also beneficial for youths themselves in improving their self-efficacy by experiencing community empowerment [11], as well as their health in later life [12]. Interestingly, the above-mentioned study in Sri Lanka [9] presented a case report in which some children involved their neighbourhood homes in the health promotion (e.g. playing games and exercising with their parents). However, no study has examined whether youths, including children, who received health education can improve the health of community adults who are not their parents or relatives. One study [13] suggested that certain segments of society may be more receptive to youth-led engagement than others, which could be applied to non-professional healthcare workers of youths. It is important to ascertain whether youths can take on the role of non-professional youth healthcare workers (or change agents, as termed in the present study), because this approach has the potential to improve the health status of many more adults than could previous approaches targeting children’s parents.

We developed an intervention program enabling youths, including school children who were members of a youth club, to act as change agents in promoting healthy lifestyles for community adults to reduce CVD risks. Under these hypotheses, the present community-based intervention study in Sri Lanka aimed to investigate the effect of youth change agents on body weight and blood pressure as primary outcomes and health-related lifestyle (e.g., physical activity and dietary intake) as secondary outcomes among community adults over a 12-month intervention.

## Methods

### Study design

This study was a cluster randomised controlled trial that incorporated youths who were members of a youth club as change agents to lower CVD risks among community adults in a semi-urban area of Colombo, Sri Lanka. The districts of Sri Lanka are divided into administrative sub-units known as Divisional Secretariat (DS) [14]. In the Colombo district, we selected one eligible DS division (Seethawaka) based on the logistical feasibility of this study. Most youth clubs are managed by the Youth Service Council, which provides vocational training and sports free of charge for youths who have registered at the Divisional Secretary office. The intervention period was set as 12 months. The effects were assessed by comparing the differences in outcome measures (i.e. changes in body weight [or body mass index, BMI] and blood pressure from pre-trial to post-trial) between selected adults living in communities assigned to intervention or control groups.

### Study settings, randomisation, and masking

**Fig. 1:**
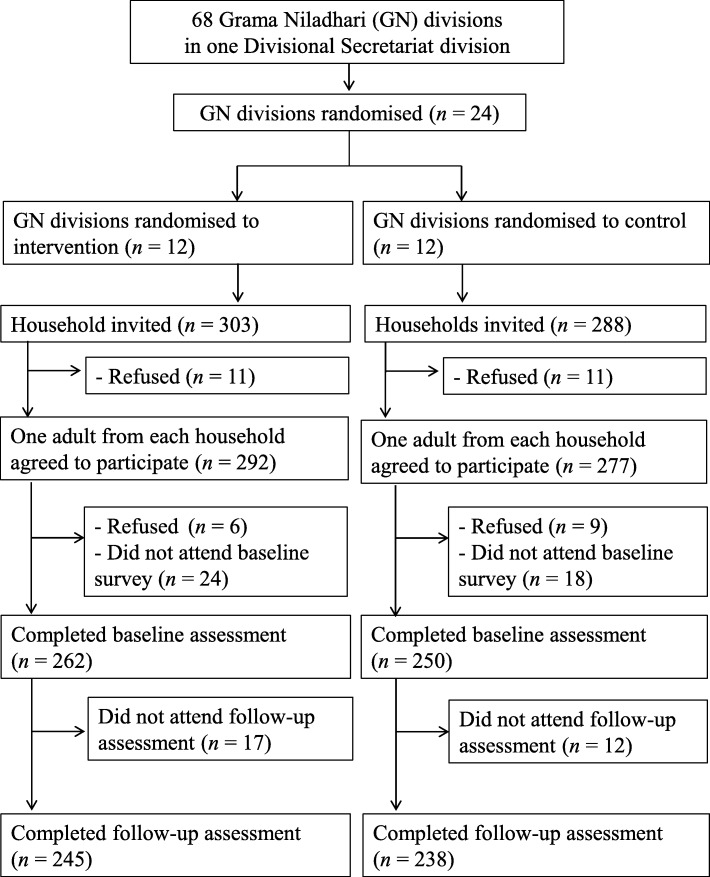
Study flow diagram

Grama Niladari (GN) division, which is a sub-unit of a DS, has the role of ensuring an administrative system at the rural level on par with the requirements of public policies [14]. As shown in Fig. 1, the DS division we selected is subdivided into 68 GN divisions [14], with one youth club on average per GN division. The required number of 24 GN divisions were then selected using a random procedure. The median (minimum, maximum) population in the 24 GN divisions was 1449 (533, 2989) [15]. We randomly allocated 12 GN divisions to the intervention and another 12 GN divisions to the comparison. Due to the nature of the intervention, it was not possible to mask the intervention allocation from the participants or youths who were involved in the intervention. However, the youths involved in outcome assessment were masked concerning the intervention allocation at both baseline and follow-up surveys.

### Intervention

#### Recruitment and training of youths

Trained facilitators of the Foundation of Health Promotion (Sri Lanka) visited 12 youth clubs and explained the goal of the project to the members as well as the procedures of health promotion activities for their community. As there were three selected GN divisions which did not have a youth club, we invited youth members through the public office of a non-selected GN division. All of the youth clubs which we approached for the intervention and the public offices mentioned above accepted the invitation and did not withdraw their acceptance. Youths who agreed to participate as change agents decided to work all week or only weekends according to their schedules with the permission of their parents. We recruited a total of 45 older adolescents and young adults aged 15–29 years who belonged to the intervention arm of youth clubs in 12 GN divisions (2–6 youths per one GN division). Prior to the intervention, facilitators trained the youths to identify behaviours linked to CVD risk factors, such as low level of physical activity, unhealthy diet, tobacco and alcohol use, and poor mental wellbeing. No youth change agents of youths received any compensation for this work.

#### Activity of youths in the community

The overall strategy was based on health promotion strategies using the models explained in previous studies conducted by us and other researchers in health promotion [9, 16]. These strategies primarily rely on the empowerment of communities to identify the determinants of undesired behaviours and community actions towards more desirable behaviours. Youths measured the body weight and blood pressure of adults once a month during the intervention period using weighing scales and an automated sphygmomanometer, which were provided by facilitators and shared with community members. The youths visited the intervention area at least twice a month to make these measurements, propose healthy lifestyle choices to adults, and encourage them to adopt improved behaviours.

Adults recorded their body weight and blood pressure as measured by the youths in a chart. Adults chose lifestyle behaviours that they would try to change under their own initiative rather than as urged by youths or facilitators. If adults showed no intention to do physical activities, youths visited their houses to invite them to the community group’s outdoor physical activities or games.

Lifestyle changes among community adults identified by youths included engagement in outdoor physical activities or games (e.g. walking, playing cricket, volleyball, or badminton with neighbours and their children), reduction in the time spent sitting and watching television, increased consumption of vegetables and fruits and reduced serving sizes of rice, measuring coconut oil for cooking by spoon, and recording dietary habits such as monthly expenses for unhealthy foods (e.g. snacks and sugar-sweetened beverages) or daily intake of rice (Additional file 1: Table S1). According to the change agents’case reports on behavioural changes made by change agents, some adults recorded their expenses for unhealthy foods such as snacks on a monthly basis, motivating themselves to cut their spending on such foods. Some adults reported reducing their excess dietary intake, such as rice servings, fast foods, and the use of cooking oil and sugar.

#### Monitoring and feedback

During the intervention, the youths were requested to make reports monitoring the progress of health promotion for their respective neighbours and report at least once per month to the facilitators, who gave advice and acknowledgement to the youths to strengthen their activity. A formal meeting was held with facilitators to review the progress of health promotion during the first 3 months of the intervention. The youths in different GNs exchanged information and acknowledgements of the health promotion to improve their skills and shared their experiences with members of different GNs. The youths then decided how they might modify their actions to improve the interest and involvement of the adults of their respective communities.

### Outcomes

#### Target

We randomly selected locations from which we would recruit participants for the outcome assessment by randomly dropping pins on road maps of the entire study area. In the selected locations, investigators visited approximately 100 houses on both sides of two or three roads. For each GN area, we made a list of 100 houses we visited based on the public house numbers. Among these 100 households, we randomly visited 23–29 households in each GN division (303 households in the intervention area and 288 households in the control area) for the outcome assessment (Fig. 1). Of these, 292 adults in the intervention group and 277 adults in the control group agreed to participate in the study. We invited one male adult or one female adult alternately from each household. Participant enrolment began in April 2016 and ended in August 2016. The baseline survey including outcome assessment were performed before change agents began their activities in the community to prevent confounding effects of the intervention.

#### Definition

The primary outcomes are the changes in body weight (or BMI) and blood pressure from baseline to follow-up surveys. The secondary outcomes are: 1) changes in physical activity (change in the proportion of adults who engaged in recommended leisure-time activities and mean difference of sedentary time), 2) changes in dietary intake (change in the proportion of adults who had an adequate intake of vegetables, fruits, snacks, and sugar-sweetened beverages), 3) changes in smoking status (change in the proportion of current smokers), and 4) changes in alcohol drinking (change in the proportion of heavy alcohol drinkers).

BMI was calculated as the body weight (kg) divided by the square of the height (m). We defined “recommended leisure-time physical activity” as over 3 hours/week of 4.5 metabolic-equivalents (METs) of moderate-intense physical activity or over 1 hour/week of 7.5 METs of vigorous physical activity, according to the review for leisure time physical activity [17]. We defined “adequate intake” as consuming two servings/day or higher for vegetables and one serving/day or higher for fruits, which roughly corresponded to the 50th to 75th percentile (1.5 to 3.5 servings/day for vegetables and 0.8 to 1.5 servings/day for fruits) of all participants at baseline. These cut-offs for vegetables and fruits are lower than the recommended values (i.e. three to five servings/day for vegetable intake and two to three servings/day for fruits) for Sri Lankans [18]. We chose cut-offs for other dietary factors (twice/day for snacks and once/day for sugar-sweetened beverages) according to the guidelines in Sri Lanka [18] and a review of sugar-sweetened beverages [19]. We decided to use two drinks per day or less for men and one drink per day or less for women as indicating a low risk of drinking level, according to the Dietary Guidelines of the United States [20].

#### Measurements

According to the WHO STEPS protocol [21], health surveys were conducted before and after the intervention. Trained youths who had not been involved in the intervention assessed the baseline and follow-up outcomes. Youths were not informed in which communities the baseline and end-line surveys were conducted. Prior to the baseline survey, youths who volunteered to perform the measurements received a half-day training course, composed of a lecture and practice, for the interview on lifestyle and physical measurement.

On the day of the survey, the youth staff members measured the body weight and height of participants in light clothing and without shoes in a public place. Weight was measured using a digital weighing scale (Rossmax WB220, Berneck, Switzerland) and recorded to the nearest 0.2 kg. Height was measured using a portable stadiometer (seca 213, Hamburg, German) and recorded to the nearest 0.1 cm. Survey staff measured the blood pressure and pulse rate of adults using an automatic blood pressure monitor (A&D UA-621, Tokyo, Japan). After participants had rested for 10 min in a quiet room, three readings were taken in the right arm at two-minute intervals with the adults in a sitting position and the arm supported at heart level. The average of the last two measurements of blood pressure was used for analysis. For those who took medication for hypertension, we assigned 140 mmHg and 90 mmHg to systolic and diastolic blood pressures, respectively, if the averaged values for each level were lower than these values.

The youth staff members interviewed participants about their health and lifestyle following a survey questionnaire. METs in hours per week of leisure-time physical activity were calculated according to the scoring system of the Global Physical Activity Questionnaire (GPAQ) [22, 23]. Additionally, we asked for the time spent on sedentary activities, including sitting and reclining [22]. Regarding the intake of vegetables and fruits, we asked for the frequency (days/week) and servings on one of those days to estimate average servings per day. We asked the frequency of intake of snacks and sugar-sweetened beverages as well, and calculated drinks (units) per day of alcohol intake among alcohol drinkers based on the frequency of intake and total drinks of alcohol beverages on one of those days.

### Sample size

The effect of the intervention was evaluated among adults living in the targeted GN divisions. Sample size was calculated to detect a 3.0 kg mean difference of body weight between the intervention group and the control group at the follow-up survey. To detect this difference with a statistical power of 80% (alpha = 0.05, two-tail), we would need a sample size of 284 adults, given a standard deviation of 9 kg of body weight, which was estimated from a previous study of mothers of school-aged children [9].

Taking into account the clustering (cluster size = 20 and intraclass correlation coefficient = 0.01, with a design effect of 1.19) [24], the sample size was 400 adults total in 20 GN divisions. To allow for a reasonable drop-out rate, we planned to recruit 480 residents from 24 GNs (i.e. 12 GNs with 240 residents in the intervention group and 12 GNs with 240 residents in the control group).

Based on the calculated sample size, 24 GN divisions out of the 68 available GN divisions were selected randomly from a list of the GN divisions. In the event of the selection of GN divisions separated by less than 3 km, they were excluded and replaced by other GN divisions to avoid any possibilities of contamination by the intervention. The 24 GN divisions were then allocated by a lottery method where 12 GN divisions were allotted to the control arm and 12 GN divisions to the intervention arm.

### Statistical analysis

The main analysis was based on the intention to treat. We performed analyses among those who completed both baseline and the follow-up surveys. We further investigated the same people pre- and post-intervention. Demographic and outcome variables were presented as mean (standard deviation [SD]) or median (interquartile rage [IQR]) for continuous data and the number (percentage) of participants for categorical data. The mean difference (SD) from baseline to the follow-up surveys was presented for continuous variables. Missing data for sedentary time at the follow-up survey (*n* = 1) were replaced with baseline data (last-observation-carried-forward [LOCF] analysis). We used two levels of multilevel models to account for the clustering of neighbours within GN divisions as a random effect in addition to individual level as a fixed effect. To take into account the difference at baseline, the model was adjusted for the baseline value of the outcome [25]. To investigate the difference in means from baseline to the follow-up, we extracted values at baseline from the values at the end of the follow-up. Finally, for the following binary outcomes (i.e. leisure-time physical activities, vegetables, fruits, snacks, sugar-sweetened beverage, current smoking, and alcohol intake), we used a multilevel logistic regression model comparing the outcomes with the low levels as a cut-off point as reference. The models were as follows:

#### Multilevel linear regression

$$ {\mathrm{outcome}}_{ij, followup}-{\mathrm{outcome}}_{ij, baseline}=\upmu +{b}_i+\alpha \cdot {\mathrm{intervention}}_{ij}+\beta \cdot {\mathrm{continuousoutcomes}}_{ij, baseline} $$

#### Multilevel logistic regression

$$ \mathrm{logitPr}\left({\mathrm{binaryoutcomes}}_{ij, followup}\right)=\upmu +{b}_i+\alpha \cdot {\mathrm{intervention}}_{ij}+\beta \cdot {\mathrm{binaryoutcomes}}_{ij, baseline} $$

where the probability of individual *i* in GN division *j* having a given outcome at the end of the follow-up (*follow up*) was modelled by intervention assignment (intervention group or control group), controlling for outcomes at baseline (*baseline*) and random intercepts for GN divisions (*b*_*i*_). The adjusted mean for the linear regression model or odds ratio (OR) for the logistic regression model and the 95% confidence interval (CI) were calculated for continuous and binary outcomes, respectively.

Subgroup analysis was performed among those who were overweight (BMI ≥ 25 kg/m^2^) or among those who had hypertension (systolic blood pressure ≥ 140 mmHg, diastolic blood pressure ≥ 90 mmHg, or receiving medical treatment for hypertension) at baseline. Two-sided *p*-values < 0.05 were regarded as statistically significant. All analyses were performed using statistical software Stata version 15.0 (StataCorp, College Station, Texas, USA).

## Results

Figure 1 shows a study flow diagram with the number of clusters and participants at each phase of the trial. From each of the 12 GN divisions in the intervention and the control group, 47 households (24 in intervention and 23 in control) were invited to the baseline survey. Five households refused to participate in the survey (2 in intervention and 3 in control). Eligible adults were invited among households (292 adults in intrevention and 277 adults in control). Of the 512 adults (262 in intervention and 250 in control) who agreed to participate and completed the baseline survey, 29 adults (17 intervention and 12 control) did not attend the follow-up survey at 12 months, leaving 483 adults (245 intervention and 238 control) for completer analyses.

Table 1 shows baseline characteristics of the study participants. The two groups were similar in terms of ethnicity, region, socioeconomic status, body weight (or BMI), blood pressure, and lifestyle variables. Mean (SD) body weight and BMI were 63.3 kg (13.7) and 25.0 kg/m^2^ (4.9) in the intervention group and 62.7 kg (12.7) and 24.9 kg/m^2^ (4.6) in the control group, respectively. The proportion of overweight (BMI 25.0–29.9 kg/m^2^) and obese (BMI ≥ 30 kg/m^2^) were nearly 35–36 and 12% in both groups. Mean (SD) systolic and diastolic blood pressure were 127.3 mmHg (18.8) and 83.4 mmHg (10.9) in the intervention group and 126.7 mmHg (20.7) and 83.6 mmHg (12.7) in the control groups.

**Table 1:** Baseline characteristics of study participants

	Intervention group	Control group
No. of participants	245	238
Age (years), mean ± SD	46.1 ± 8.1	44.8 ± 8.2
Women	130 (53.1)	124 (52.1)
Ethnicity		
Sinhalese	234 (95.5)	226 (95.0)
Tamil	10 (4.1)	11 (4.6)
Muslim or others	1 (0.4)	1 (0.4)
Religion		
Buddhism	218 (89.0)	218 (91.6)
Hindu	7 (2.9)	10 (4.2)
Roman Catholic/Christian	19 (7.8)	10 (4.2)
Islam	1 (0.4)	0 (0)
Education attainment		
Primary level (grade 1–5)	34 (13.9)	29 (12.2)
Junior high school	145 (59.2)	150 (63.0)
High school or higher	66 (26.9)	59 (24.8)
Household income (Rp/month)^a^		
≤40,000	200 (81.6)	200 (84.0)
40,001–60,000	32 (13.1)	25 (10.5)
≥60,001	13 (5.3)	13 (5.5)
Current workers	123 (50.2)	137 (57.6)
Current diseases		
Type 2 diabetes	42 (17.1)	39 (16.4)
Dyslipidemia	25 (10.2)	33 (13.9)
Hypertension	55 (22.5)	47 (19.8)
BMI categories		
<18.5 kg/m^2^	21 (8.6)	20 (8.4)
18.5–24.9 kg/m^2^	110 (44.9)	103 (43.3)
25.0–29.9 kg/m^2^	85 (34.7)	86 (36.1)
≥30 kg/m^2^	29 (11.8)	29 (12.2)
Primary outcomes		
Body weight (kg), mean ± SD	63.3 ± 13.7	62.7 ± 12.7
BMI (kg/m^2^), mean ± SD	25.0 ± 4.9	24.9 ± 4.6
Systolic blood pressure (mmHg), mean ± SD	127.3 ± 18.8	126.7 ± 20.7
Diastolic blood pressure (mmHg), mean ± SD	83.4 ± 10.9	83.6 ± 12.7
Secondary outcomes		
Physical activity		
Leisure-time physical activities, recommended level^b^	47 (19.2)	43 (18.1)
Sedentary times (minutes/day), median (IQR)	180 (120, 300)	180 (120, 300)
Dietary intake		
Vegetables, ≥ two servings/day	83 (33.9)	92 (38.7)
Fruits, ≥ one serving/day	84 (34.3)	86 (36.1)
Snacks, ≥ twice/day	90 (36.7)	91 (38.2)
Sugar-sweetened beverages, ≥ once/day	20 (8.2)	15 (6.3)
Current smoking	38 (15.5)	36 (15.1)
Alcohol intake, > low risk of drinking level^c^	11 (16.9)	18 (26.9)

*Rp* Sri Lankan Rupees, *BMI* body mass index, *SD* standard deviation, *IQR* inter-quartile range

Data are numbers (percentages) unless otherwise indicated

^a^The rate of Sri Lankan Rupees into US Dollar (USD) in 7th of July 2017 was 1 Rp = 0.0067 USD

^b^Recommended leisure-time physical activity was defined over 3 hours/week of 4.5 metabolic-equivalents (METs) of moderate-intense physical activity or over 1 hour/week of 7.5 METs of vigorous-intense physical activity

^c^low risk of drinking level was two drinks/day for men and one drink/day for women

Table 2 shows the differences in the outcome variables between the intervention and the control groups at the 12-month follow-up survey. With respect to the primary outcomes at the follow-up, the intervention group had a significantly lower mean (SD) body weight (61.8 kg [12.7]) and BMI (24.4 kg/m^2^ [4.4]) than did the control group (body weight 64.0 kg [12.8] and BMI 25.5 kg/m^2^ [4.7]); the mean difference (95% CI) upon comparing the intervention with the control group was − 2.83 kg (− 3.31 to − 2.35) for body weight and − 1.12 kg/m^2^ (− 1.32 to − 0.94) for BMI (difference between groups, *p* < 0.001). In a subgroup analysis among adults who were overweight (BMI ≥ 25 kg/m^2^) at baseline, we observed a more pronounced difference; the mean (95% CI) difference between the intervention and control groups was − 3.69 kg (− 4.48 to − 2.90) for body weight and − 1.50 kg/m^2^ (− 1.80 to − 1.20) for BMI (difference between groups, *p* < 0.001) (Appendix Table 1).

**Table 2:** Effect of intervention on primary and secondary outcomes at 12th-month follow-up

	Intervention group (*n* = 245)		Control group (*n* = 238)		Between-group difference at follow-up^c^	
	Mean ± SD, median (IQR)^+^, or number (%) at the end of the follow-up	Mean ± SD of change from baseline^a^	Mean ± SD, median (IQR)^+^, or number (%) at the end of the follow-up	Mean ± SD of change from baseline^a^	Difference in means^b^ or OR (95% CI)	*p*-value
Primary outcomes						
Body weight (kg)	61.8 ± 12.7	−1.51 ± 3.18	64.0 ± 12.8	1.36 ± 2.30	− 2.83 (− 3.31, − 2.35)^b^	< 0.001
BMI (kg/m^2^)	24.4 ± 4.4	− 0.59 ± 1.27	25.5 ± 4.7	0.54 ± 0.95	− 1.12 (− 1.32, − 0.94)^b^	< 0.001
Systolic blood pressure (mmHg)	127.1 ± 18.9	0.44 ± 15.4	128.4 ± 18.1	1.11 ± 13.4	−0.88 (− 3.18, 1.42)^b^	0.45
Diastolic blood pressure (mmHg)	84.0 ± 10.7	0.44 ± 10.6	85.4 ± 10.1	1.01 ± 9.20	−0.94 (− 2.64, 0.77)^b^	0.28
Secondary outcomes						
Physical activity						
Leisure-time physical activities, recommended level^d^	31 (12.7)		20 (8.4)		1.58 (0.84, 2.96)	0.15
Sedentary times (minutes/day)	180 (120, 300)^+^	3.33 ± 180	180 (120, 300)^+^	5.27 ± 140	1.16 (− 28.0, 30.3)^b^	0.94
Dietary intake						
Vegetables, ≥ two servings/day	106 (43.3)		92 (38.7)		1.24 (0.79, 1.94)	0.35
Fruits, ≥ one serving/day	112 (45.7)		82 (34.5)		1.71 (1.10, 2.65)	0.02
Snacks, ≥ twice/day	52 (21.2)		107 (45.0)		0.32 (0.21, 0.48)	< 0.001
Sugar-sweetened beverages, ≥ once/day	14 (5.7)		15 (6.3)		0.86 (0.35, 2.09)	0.74
Current smoking	29 (11.8)		29 (12.2)		0.86 (0.32, 2.31)	0.77
Alcohol intake, > low risk of drinking level^e^	9 (11.8)		17 (22.1)		0.50 (0.15, 1.71)	0.27

*BMI* body mass index, *SD* standard deviation, *IQR* inter-quartile range, *CI* confidence interval

^a^Change from baseline = outcome values at the end of the follow-up – outcome values at baseline

^b^The analysis of ‘difference in means’ was used the calculated outcome of ‘change from baseline’

^c^Multilevel linear regression for continuous outcomes and multilevel logistic regression for binary outcomes, with Grama Niladari divisions as the cluster variable and adjustment for each outcome variable at baseline

^d^Recommended leisure-time physical activity was defined over 3 hours/week of 4.5 metabolic-equivalents (METs) of moderate-intense physical activity or over 1 hour/week of 7.5 METs of vigorous-intense physical activity

^e^low risk of drinking level was two drinks/day for men and one drink/day for women

Blood pressure, which was one of the primary outcomes, did not significantly change from the baseline to the follow-up in either group. There was no significant intervention effect on blood pressure; the mean differences (95% CI) between the intervention and control groups in systolic and diastolic blood pressure were − 0.88 (− 3.18, 1.42) and − 0.94 (− 2.64, 0.77), respectively. Among those who had hypertension at baseline, there was also no intervention effect (Additional file 2: Table S2).

With regard to health-related lifestyle in secondary outcomes, there were no statistically significant differences in the odds of being engaged in recommended levels of leisure-time physical activities between the two groups at the follow up. The intervention group showed significantly higher odds of consuming ≥ one serving/day of fruits (OR 1.71, 95% CI 1.10–2.65) and significantly lower odds of consuming ≥ twice/day snacks (OR 0.32, 95% CI 0.21–0.48) than the control group. There was no significant difference in the consumption of vegetables or sugar-sweetened beverages between the two groups.

## Discussion

In this randomised controlled trial that enlisted youths as change agents to lower CVD risks among community adults in Sri Lanka, the intervention group showed a statistically significantly greater reduction in body weight than the control group. As regards diet, the proportion of adults who consumed the recommended levels of fruits was significantly higher and the proportion of adults who consumed snacks frequently was significantly lower in the intervention group than in the control group. There was no significant difference in blood pressure between the two groups. To our knowledge, this is the first study to report the effectiveness of the intervention by youths for the health of community adults.

The present findings regarding the effect of the intervention on body weight are in line with our previous study in Sri Lanka [9], showing a larger weight reduction in mothers who received support from their school-aged children to identify and improve determinants of health in their family; the mean difference (95% CI) of body weight between the intervention with the control group was −2.49 kg (−3.38, −1.60). In contrast, there was no intervention effect on body weight observed in a Brazilian study [8], where school children received an educational program for CVD prevention and asked parents to modify the lifestyle of the whole family. Such different results for body weight reduction may be attributed to the difference in outcome settings and strategy of the intervention. In the present and the previous study in Sri Lanka [9], body weight was one of the primary outcomes and the intervention was designed to modify the lifestyles of adults with an emphasis on weight control, as we will discuss in the next paragraph in greater detail. In the Brazilian study [8], the education for the children covered nutrition, exercise, and smoking to reduce the overall CVD risk profile of parents, but did not specifically address weight reduction. Moreover, the current and previous study in Sri Lanka [9] actively intervened in the lifestyle of adults through children or youths, whereas in the Brazilian study [8] children were the target of education and were asked not to feel responsible for lifestyle modifications of their family.

The weight reduction observed in the present study may be primarily attributed to the improvements in diet. The intervention group consumed snacks less frequently than the control group after the intervention. According to the case reports reported by youths, some adults tried to cut their spending on unhealthy foods and reduce their excess dietary intake by recording their dietary habits. The monthly plotting of body weight in the chart might facilitate adults in avoiding energy dense foods and eating too much. We also observed a significant increase in fruits intake in the intervention group. There is evidence suggesting a protective role of fruit fibre against obesity [26]. The replacement of snacks and other energy dense foods with fruits might be effective in weight management [27].

Turning to leisure-time physical activity, the youths encouraged adults to do some exercise, such as outside activities or games with other adults and/or their children together. According to the reports from the youth, adults living in about 60% of the intervention GNs did physical exercise enthusiastically with the support of the youths. In spite of these promotions, the prevalence of adults who engaged in the recommended level of leisure-time physical activity in the intervention group was not significantly higher than that in the control group after the intervention. One reason for this finding could be the insufficient ability of the questionnaire we used to detect moderate changes of the physical activity.

We observed no intervention effect on blood pressure. In contrast, a significant reduction in blood pressure was reported in a Chinese study [10] in which students were educated to deliver salt reduction message to their parents. Unlike the Chinese study [10], the present study did not specifically address salt reduction, whose effect on lowering blood pressure is convincing [28]. Future studies for the prevention of hypertension should incorporate salt reduction as a major component of the intervention, especially in countries with high salt consumption.

It remains to be determined whether the present findings are applicable to other settings and how the present approach of community health promotion by youth club could be scaled up in a wider context. Besides the presence of active youth club or similar association in the community, it is necessary to develop a facilitator-support system from which youths can receive comprehensive guidance. Youths should be guided to promote their awareness, understanding, and tolerance of other people, cultures, and societies so that they can provide standardized and high-quality intervention universally as change agents.

The current study is strengthened by its cluster randomised controlled design, preventing the influence of measured and unmeasured confounders. The study limitations also warrant mention. First, lifestyle factors such as physical activity and dietary habits were self-reported, and misclassification due to inaccurate responses might thus have diluted the effect of the intervention. Second, lifestyles were assessed by interviews, raising a concern of interviewer bias. However, the interviewers were blinded to the intervention allocation. Therefore, such a possibility would be low. Third, there were variations across the intervened communities in the type of activities and frequency of community visits of youths according to youths’ skills and motivations. The effect of intervention may be attenuated due to the inclusion of communities with low levels of health promotion activities. Fourth, we cannot clarify the long-term effect of this program beyond 12 months. Fifth, although this study applied an intention-to-treat approach [29], we excluded refusals and non-attendance at the follow-up survey from all randomized subjects (303 subjects in the intervention group and 288 subjects in the control group). Sixth, although most of the adults received support from the youths to improve their health behaviours, we did not record them. Therefore, there was a possibility that there were adults who did not receive a direct approach from youths, whereas we expected that adults might receive not only a direct approach from change agents but also a spillover effect [30] of the change agents on health behaviours among adults in the community. Finally, it remains unclear whether this type of program is generalisable for the following reasons: 1) the present study was performed in a semi-urban area of Colombo, 2) we selected one DS division based on logistical feasibility before the randomization setting, and 3) there was a possibility of some influence of the health survey, which was conducted before the trial, on health behaviours for both the intervention group and the control group, although we could not assess whether this health survey attenuated or promoted the effect of change agents of youth.

## Conclusions

The present randomised controlled trial in Sri Lanka showed that a 12-month program in which youths were trained to act as change agents in their community was effective in decreasing body weight but not blood pressure among adults. A health promotion to improve diets performed by youths might be effective in body weight reduction in adults.

## Additional files

Additional file 1: **Table S1.** Examples of health behaviours of adults implemented in the intervention group. (DOCX 17 kb)

Additional file 2: **Table S2.** Effect of intervention on body weight for people with overweight and on blood pressure for people with hypertension. (DOCX 19 kb)

## Data Availability

The datasets used and/or analysed during the current study are available from the corresponding author on reasonable request.

## Abbreviations

BMI: body mass index
CI: confidence interval
DS: divisional secretariat
GN: grama niladari
METs: metabolic equivalents
NCD: non-communicable disease
SD: standard deviation
