# Correction to: The effect of youths as change agents on cardiovascular disease risk factors among adult neighbours: a cluster randomised controlled trial in Sri Lanka

**DOI:** 10.1186/s12889-019-8096-z

**Published:** 2020-01-13

**Authors:** Nadeeka Chandraratne, Miwa Yamaguchi, Susantha Indrawansa, Nalika Gunawardena, Keisuke Kuwahara, Zobida Islam, Yohei Kawasaki, Tetsuya Mizoue, Diyanath Samarasinghe

**Affiliations:** 1 Ministry of Health, Nutrition and Indigenous Medicine, Suwasiripaya, No. 385, Rev. Baddegama Wimalawansa Thero Mawatha, Colombo, 10 Sri Lanka; 2 Department of Epidemiology and Prevention, National Centre for Global Health and Medicine, 1-21-1 Toyama, Shinjyuku, Tokyo, 162-8655 Japan; 3 The Foundation for Health Promotion, No.21/1 Kahawita Road, Attidiya, Dehiwala, Western Province 10350 Sri Lanka; 4 World Health Organisation Country Office for Sri Lanka, 5 Anderson Road, Colombo, Sri Lanka; 5 Teikyo University Graduate School of Public Health, Tokyo, Japan; 6 Biostatistics Section, Clinical Research Center, Chiba University Hospital, 1-8-1 Inohana, Chuo-ku, Chiba-shi, Chiba, 260-8677 Japan; 7 Department of Psychological Medicine, University of Colombo, Colombo, Sri Lanka

**Correction to: BMC Public Health (2019) 19:893**

**https://doi.org/10.1186/s12889-019-7142-1**

It was highlighted that in the original article [1] the selection process was not described clearly enough to avoid confusion under the heading of the Target of Outcomes in the Methods section. The authors thus decided to modify some parts in order to render more clear the relationship between household and adult. This Correction articles shows the incorrect and correct statement.

**Original**

Among these 100 households, we randomly visited 23–29 households in each GN division (303 households in the intervention area and 288 households in the control area) for the outcome assessment (Fig. 1). Of these, 292 adults in the intervention group and 277 adults in the control group agreed to participate in the study. We invited one male adult or one female adult alternately from each household.

**Modified**

Of these 100 households in each GN division, we randomly selected and visited 23–29 households (303 households in the intervention area and 288 households in the control area) for the outcome assessment (Fig. 1). We invited one male adult or one female adult alternately from each household. Of those invited, 292 adults in the intervention group and 277 adults in the control group agreed to participate in the study.
