# Supplementary material for: The effect of youths as change agents on cardiovascular disease risk factors among adult neighbours: a cluster randomised controlled trial in Sri Lanka
Source: BMC Public Health. 2019 Jul 8;19:893. doi: 10.1186/s12889-019-7142-1 (PMC6613264; doi:10.1186/s12889-019-7142-1)
Supplement: Supplementary file 1 — Table S1. Examples of health behaviours of adults implemented in the intervention group. (DOCX 17 kb) [file 12889_2019_7142_MOESM1_ESM.docx]

**Additional file 1**

**Table S1** Examples of health behaviours of adults implemented in the intervention group

| **Physical activities** | **Dietary pattern** | **Record in the chart** |
| --- | --- | --- |
| Play cricket with children  Play cricket, volleyball, or badminton with their children and/or neighbours  Swim in the lake  Climb hills with their children and/or neighbours  Walk with neighbours at the playground  Reduce time sitting in front of the television  Spend more time in the home garden  Do daily housework faster than usual  Sing songs with their families and/or neighbours in the evening | Reduce rice servings and increase vegetables and fruits  Reduce intake of sugar and daily quantity of sweet tea  Reduce intake of fast food  Reduce the use of salt in rice  Use smaller plates for serving food  Stop watching television while having meals  Measure daily use of coconut oil by using a spoon  Reduce purchases of and save money on coconut oil, sugar, milk powder, and flour | Monthly expenditures on unnecessary foods  Monthly expenditures on coconut oil, sugar, milk powder, and flour  Monthly body weight change  Monthly blood pressure |
