# Supplementary material for: The effect of youths as change agents on cardiovascular disease risk factors among adult neighbours: a cluster randomised controlled trial in Sri Lanka
Source: BMC Public Health. 2019 Jul 8;19:893. doi: 10.1186/s12889-019-7142-1 (PMC6613264; doi:10.1186/s12889-019-7142-1)
Supplement: Supplementary file 2 — Table S2. Effect of intervention on body weight for people with overweight and on blood pressure for people with hypertension. (DOCX 19 kb) [file 12889_2019_7142_MOESM2_ESM.docx]

**Additional file 2**

**Table S2** Effect of intervention on body weight for people with overweight and on blood pressure for people with hypertension

|  | Intervention group | | |  | Control group | | |  | Between-group difference at  follow-up^†^ | |
| --- | --- | --- | --- | --- | --- | --- | --- | --- | --- | --- |
|  | n | Mean ± SD  at the end of the follow-up | Mean ± SD of change from baseline |  | n | Mean ± SD  at the end of the follow-up | Mean ± SD of change from baseline |  | Difference in means (95 % CI) | *p*-value |
| People with overweight^‡^ |  |  |  |  |  |  |  |  |  |  |
| Body weight (kg) | 114 | 71.1 ± 10.7 | -2.56 ± 3.03 |  | 115 | 72.4 ± 10.8 | 1.19 ± 2.64 |  | -3.69 (-4.48, -2.90) | < 0.001 |
| BMI (kg/m^2^) | 114 | 28.1 ± 3.3 | -1.04 ± 1.25 |  | 115 | 29.1 ± 3.2 | 0.48 ± 1.11 |  | -1.50 (-1.80, -1.20) | < 0.001 |
| People with hypertension^§^ |  |  |  |  |  |  |  |  |  |  |
| Blood pressure |  |  |  |  |  |  |  |  |  |  |
| Systolic (mmHg) | 108 | 140.5 ± 17.8 | -2.85 ± 18.0 |  | 110 | 142.3 ± 15.4 | 2.09 ± 17.2 |  | -3.18 (-7.05, 0.68) | 0.11 |
| Diastolic (mmHg) | 108 | 92.0 ± 9.2 | -1.37 ± 12.8 |  | 110 | 93.6 ± 7.1 | -1.37 ± 12.8 |  | -1.90 (-4.33, 0.54) | 0.13 |

BMI body mass index, SD standard deviation, CI confidence interval

Change from baseline = outcome values at the end of the follow-up – outcome values at baseline

^†^Multilevel linear regression for continuous outcomes and multilevel logistic regression for binary outcomes, with Grama Niladari divisions as the cluster variable and adjustment for each outcome variable at baseline.

^‡^Overweight was defined BMI ≥ 25 kg/m^2^.

^§^Hypertension was defined systolic blood pressure ≥ 140mmHg, diastolic blood pressure ≥ 90 mmHg, or receiving medical treatment for hypertension.
